# 

**Published:** 1888-01

**Authors:** 


CONTENTS, JANUARY, 1888, (VOL. III, NO. VII.)

ORIGINAL COMMUNICATIONS.—Cocaine in Urethral Surgery; A. F. Sampson, M. D., Galveston................252
Septic Poison; L. D. Hill, M. D., Webberville, Texas....257
Etiology of Contagious Diseases; J. W. McLaughlin, M. D., Austin........................................266
CORRESPONDENCE.—Prof. Hotz' Operation for Trichiasis—Letter from Dr. Yoakum, of Greenville, Tex.............274
The Malarial Germ; Letter from Dr. Felder, of Cleburne, and Report of Microscopic Examination of Spores, by Dr. Menger........................................276
FOREIGN CORRESPONDENCE.—Letter from Our Own Paris Correspondent........................................279
SOCIETY NOTES.—Texas State Medical Association; Annual Address of the President..............................283
Physicians' Mutual Benefit Association of Texas; Statement of the Secretary; Operations and Disbursements........290
Letter and Receipt from Mrs. Kilpatrick................248
List of Members who Paid Assess. No. 4.............. 285-7
Texas State, Williamson County, Travis County, East Texas, East Line, and North Texas Med. Societies......288-9
EDITORIAL.—Taxation Without Representation ......... 291
Death of Dr. S. F. Starley..............................293
Meeting of Physicians of Tyler, and Resolutions on Death of Dr. Starley........................................294
Dr. McLaughlin's Paper..................................288
BIBLIOGRAPHY.—Books and Pamphlets Received...........296
MEDICAL NEWS AND MISCELLANY...........................300
ADVERTISERS' NOTICES...................................306

